# Depression and anxiety among patients with psoriasis: A correlation with quality of life and associated factors

**DOI:** 10.1016/j.jtumed.2021.02.008

**Published:** 2021-03-16

**Authors:** Raishan S. Bakar, Sharifah Z.S. Jaapar, Afiq F. Azmi, Yeoh C. Aun

**Affiliations:** a Department of Psychiatry, School of Medical Sciences, Universiti Sains Malaysia, Kelantan, Malaysia; b Department of Psychiatry Hospital Sultanah Bahiyah, Alor Setar, Kedah, Malaysia; c Dermatology Department, Hospital Sultanah Bahiyah, Alor Setar, Kedah, Malaysia

**Keywords:** الصدفية, الاكتئاب, القلق, نوعية الحياة, خلل شحميات الدم, Anxiety, Depression, Dyslipidaemia, Psoriasis, Quality of life

## Abstract

**Objectives:**

Psoriasis is a chronic, immune-mediated illness that primarily affects the skin, nails and joints. This illness may predispose people to mental disorders such as depression and anxiety. This study aims to determine the prevalence of depression and anxiety in patients with psoriasis and their correlation with quality of life and associated factors.

**Methods:**

This cross-sectional study involved 174 patients with psoriasis at the dermatology clinic of Hospital Sultanah Bahiyah, Alor Setar. All patients were instructed to complete four sets of questionnaires relating to: sociodemographic profile, clinical characteristics of illness, the validated Malay version of Hospital Anxiety and Depression Scale (HADS) and the Malay validated version of Dermatology Life Quality Index (DLQI). Data were analysed using a descriptive analysis and correlational and multiple logistic regression analyses.

**Results:**

We have found that 8.5% patients had depressive and 16.9% had anxiety symptoms. Multiple logistic regression analysis showed that higher DLQI scores, presence of lower limbs' lesions and dyslipidaemia were associated with depression in the sampled population.

**Conclusion:**

This study has shown that the cohort with psoriasis exhibited notable symptoms of depression and anxiety. This emphasises the need for the assessment of anxiety and depression among patients with psoriasis as these symptoms predict poor quality of life. Such correlation of psoriasis with anxiety and depression essentially leads to psychological sequelae. Affected psoriasis patients need appropriate intervention. Our study paves the way for further research by involving other underlying constructs such as perceived body image and stigma.

## الملخص

**أهداف البحث:**

الصدفية هي مرض مزمن، بسبب المناعة يؤثر في المقام الأول على الجلد والأظافر والمفاصل. قد يجعل هذا المرض المرضى المصابين به عرضة للاضطرابات النفسية مثل الاكتئاب والقلق. تهدف هذه الدراسة إلى تحديد انتشار الاكتئاب والقلق في المرضى الذين يعانون من الصدفية وارتباط ذلك بجودة الحياة والعوامل المرتبطة به.

**طرق البحث:**

شملت هذه الدراسة المقطعية ١٧٤ مريضا بالصدفية في عيادة الأمراض الجلدية في مستشفى سلطانة بهية، آلور سيتار. وصدرت تعليمات إلى جميع المرضى بملء أربع مجموعات من الاستبانات؛ الملامح الاجتماعية الديموغرافية، والخصائص السريرية للمرض، والنسخة الماليزية المحققة من مقياس المستشفى للقلق والاكتئاب والنسخة الماليزية المحققة من مؤشر جودة الحياة للأمراض الجلدية. تم تحليل البيانات باستخدام تحليل وصفي وتحليل الترابط والتراجع اللوجستي المتعدد.

**نتائج:**

لقد وجدنا أن ٨.٥٪ من المرضى يعانون من الاكتئاب و١٦.٩٪ كان لديهم أعراض القلق. أظهر تحليل التحوف اللوجستي المتعدد أن ارتفاع درجات النسخة الماليزية المحققة من مؤشر جودة الحياة للأمراض الجلدية، ووجود آفات الأطراف السفلية، وكان خلل شحميات الدم مرتبط بالاكتئاب في العينة التي تم أخذ عينات منها.

**الاستنتاجات:**

أظهرت هذه الدراسة أن عينة الدراسة المصابة بالصدفية أظهرت أعراضا ملحوظة من الاكتئاب والقلق. تؤكد هذه النتيجة على الحاجة إلى تقييم القلق والاكتئاب بين المرضى الذين يعانون من الصدفية لأن هذه الأعراض تتوقع سوء نوعية الحياة. مثل هذا الارتباط بين الصدفية والقلق والاكتئاب يؤدي أساسا إلى عواقب نفسية. ويحتاج مرضى الصدفية المتضررين إلى التدخل المناسب. تمهد أبحاثنا الطريق لمزيد من البحث من خلال إشراك الثوابت الأساسية الأخرى مثل صورة الجسم المتصورة ووصمة العار.

## Introduction

Psoriasis is a chronic, immune-mediated systemic disease that primarily affects skin, nail and joints. The prevalence ranges between 0.09% and 11.3% based on various studies.^1^ In Malaysia, there is no population-based epidemiological research on psoriasis, but the prevalence is estimated to range between 2% and 6% based on dermatology clinic attendees.^2^ The illness is caused by an interplay of several components such as genetics, environmental and the immune system.

Besides the physical symptoms of this illness, such as itchiness and pain, psoriasis also causes disfiguration and disability that would overall negatively impact the quality of life for those suffering from it. Psoriasis is also associated with an increased risk of developing other comorbidities such as cardiovascular and other non-communicable diseases. The associated comorbidities include psychiatric illnesses. Compared with the general population, patients with psoriasis have 40–90% more psychological comorbidities, manifested by high levels of anxiety, pathological levels of worrying, depression and suicidal ideation.^3^

There are several theories from biological, psychological and social aspects that link psoriasis with mental well-being. Biologically, the relationship between the two is theorised to be mediated through the hypothalamic-pituitary-adrenal (HPA) axis, which is dysregulated in times of stress with a net result of an increase in pro-inflammatory mediators. Furthermore, various studies have shown that mood disorders such as depression are interlinked with the immune system, evidenced by an increase in biological markers such as inflammatory cytokines and the presence of chronic inflammation.^4^

The physical signs of the disease are alarming to the public, more than other chronic diseases. A cultural belief of lack of self-hygiene associated with skin diseases and their contagiousness contribute to negative public perceptions.^5^ A negative stigmatisation leads to social discrimination and misconception. Moreover, at the individual level, the person's ideal body image may lead to embarrassment, anxiety and depression.

Besides the psychological sequelae of psoriasis, the quality of life is negatively affected in the context of daily activities, occupational function, and social and sexual relationships, which may in turn contribute to continuous stress and difficulty in achieving remission. The factors associated with depression and anxiety include higher Psoriasis Area and Severity Index (PASI) scores,^6^, ^7^, ^8^ feelings of helplessness and perceived lack of social support.^9^

Studies conducted in Malaysia are mostly related to stress, which is the main triggering factor for psoriasis exacerbations.^10^^,^^11^ Thus, there is still a lack of data regarding the prevalence of depression and anxiety among patients with psoriasis in the local population, which is the main objective set out for this research. It also aims to find any factors associated with depression and anxiety in this population.

## Materials and Methods

A cross-sectional study was conducted on 174 participants with psoriasis at the outpatient Dermatology Clinic in Hospital Sultanah Bahiyah, Alor Setar between February and April 2019. Convenience sampling method was applied in this research. Participants were 18 and above, literate in Malay language, and had not been diagnosed with any mental illness or cognitive impairment.

The self-administered questionnaires included questions on sociodemographic characteristics, clinical profile of participants with psoriasis, the Malay version of the Hospital Anxiety and Depression Scale (HADS), and the Malay version of Dermatology Life Quality Index (DLQI). Demographic characteristics of the participants included basic information such as age, race, gender, marital status, educational qualification, employment status and household income.

Participants were asked to identify the type of psoriasis they were diagnosed with, duration of illness, any previous history of hospitalisation for a psoriasis, presence or absence of psoriatic arthropathy and any underlying co-morbidities. To find any association between current anatomical site of psoriatic lesions with depressive and anxiety symptoms, they were also asked to identify the former. To clarify, some information was counter-checked with the clinical records in the hospital computer system.

The Hospital Anxiety and Depression Scale (HADS) is a self-administered questionnaire that was developed by Zigmond and Snaith. It contains 14 items, of which seven correspond to depression (HADS-D) and the remaining seven to anxiety (HADS-A). HADS was originally developed as a scale to assess depressive and anxiety symptoms of patients in surgical and medical settings, but has since been evaluated and validated for usage in different medical, psychiatric and non-medical populations. Participants of this study were asked to score each item from 0 to 3 (four-point Likert scale), giving a total of 21 each for anxiety and depression subscales. This study utilises the translated Malay version of the original tool, which suggested a cut-off point of 8/9 for anxiety and depression.^12^ This version of HADS showed good sensitivity and specificity (sensitivity 90.0% and specificity 86.2% for anxiety; sensitivity 93.2% and specificity 90.8% for depression) and therefore, is a valid instrument for use in the Malaysian population.^12^

The Dermatology Life Quality Index (DLQI) was developed by Andre Y Finlay and Gul Karim Khan from 1990 to 1994 to measure the impact of skin illnesses on a patient's life in the previous week. It contains 10 questions, grouped into six subscales: symptoms and feelings, daily activities, leisure, work and school, personal relationships and treatment. For each item, the participants were asked to score ‘0’ for ‘not at all’ or ‘not relevant’, ‘1’ for ‘A little’, ‘2’ for ‘A lot’ and ‘3’ for ‘very much’. The scale yields a maximum score of 30. This study uses the validated Malay version of DLQI, which is available from the website of the Department of Dermatology, Cardiff University, Cardiff, United Kingdom. The instrument has been used to measure the quality of life of patients with psoriasis in the National Dermatology Registry (DermReg), Malaysia and has been utilized widely in many countries. The permission to use this questionnaire was granted by Prof Finlay.

Data entry and analysis were done using SPSS version 24.0. Descriptive analysis was carried out for sociodemographic profile and clinical characteristics of participants. Measurement of mean scores of HADS and DLQI were used to ascertain the prevalence of depression and anxiety as well as the quality of life's category. The categories are based on their cut-off point value. To determine factors that are associated with depression and anxiety, multiple logistic regression was used. Independent variables were initially subjected to simple logistic regression analysis and variables that have p-value of < 0.25 in the initial analysis or those judged to be clinically important were entered in multivariate analysis. Both univariate and multivariate analyses were performed in this study for all the independent variables.

## Results

A total of 174 participants enrolled in this study. There were more males (94, 54% than females (80, 46%). The majority of the patients are Malay (140, 80.5%), followed by Chinese (27, 15.5%), Indians (6, 3.4%) and that from another ethnic background (1, 0.6%). The majority of the participants have a lower educational background (completed secondary school and below), adding up to 124 (71.2%), followed by a higher educational background (50, 28.8%). As for household income, most of the participants (140, 80.5%) are categorised into the B40, lowest income group with a total household income of RM 3,860 and below.

In terms of the type of psoriasis, majority of the participants (164, 94.3%) were diagnosed to have plaque psoriasis and only a small number had guttate (2, 1.1%), palmoplantar (2, 1.1%), pustular (1, 0.6%) and erythrodermic (5, 2.9%) psoriasis. There were 59 (33.9%) participants who were diagnosed to have psoriatic arthropathy. For the anatomical site of psoriatic lesions, those on the lower limbs were reported most commonly by participants (137, 78.7%), followed by scalp lesions (128, 73.6%), upper limbs (126, 72.4%), trunk (125, 71.8%), facial (54, 31%) and palm (27, 15.5%) lesions. Diabetes mellitus (43, 24.7%), hypertension (55, 31.6%), and dyslipidaemia (53, 30.5%) were among the comorbidities diagnosed among the participants (See Table 1).

**Table 1:** Descriptive statistic on sociodemographic and clinic data (n = 174).

Variable	n (%)	Mean (SD)
Age (Years)		46.4 (15.14)
Gender		
Male	94 (64)	
Female	80 (46)	
Race		
Malay	140 (80.5)	
Non-Malay	34 (19.5)	
Education Background		
Lower Education and below	124 (71.2)	
Higher education	50 (28.8)	
Employment Status		
Employed	103 (59.2)	
Unemployed	71 (40.8)	
Marital Status		
Married	124 (71.3)	
No spouse/partner	50 (28.7)	
Household Income		
Above RM 3,860	34 (19.5)	
Below RM 3,860	140 (80.5)	
Type of Psoriasis		
Non Plaque	10 (5.7)	
Plaque	164 (94.3)	
History of Admission due		
No	155 (89.1)	
Yes	19 (10.9)	
Psoriatic Arthropathy		
No	115 (66.1)	
Yes	59 (33.9)	
Scalp Lesions		
No	46 (26.4)	
Yes	128 (73.6)	
Facial Lesions		
No	120 (69)	
Yes	54 (31)	
Upper Limbs' Lesions		
No	48 (27.6)	
Yes	126 (72.4)	
Medical Co-morbidities		
Diabetes mellitus	43 (24.7)	
Hypertension	55 (31.6)	
Dyslipidaemia	53 (30.5)	

In this study, the prevalence of participants with depressive symptoms is 15 (8.5%) with a HADS-D mean score of 3.7 (SD 3.253), whereas for anxiety, this study shows that 30 (16.9%) of participants had anxiety symptoms, with a HADS-A mean score of 4.22 (SD 3.659). Overall, 78 (44.9%) of the participants had a DLQI score of more than 10, indicating severe impairment in their quality of life (Table 2). Of this, 45 (25.9%) had reported moderate effect of psoriasis on their quality of life, 28 (16.1%) were very largely affected by psoriasis and 5 (2.9%) indicated that their quality of life was extremely affected by psoriasis.

**Table 2:** Dermatology Life Quality Index Score.

DLQI score category		n (%)
No effect on patient's life (0–1)		28 (16.1)
Small effect on patient's life (2–5)		68 (39)
Moderate effect on patient's life (6–10)		45 (25.9)
Very large effect on patient's life (11–20)		28 (16.1)
Extremely large effect on patient's life (21–30)		5 (2.9)

Pearson's correlation coefficients were measured to ascertain the linear relationship between HADS-D and HADS-A subscales with DLQI score. There is positive correlation between HADS-D and DLQI (r = 0.421, p-value <0.001) and between HADS-A and DLQI (r = 0.465, p-value <0.001).

Simple logistics regression for initial model was performed and the variables that met the initial screening criterion of p < 0.25 were then regressed using multiple logistic regression using forward, backward and stepwise methods.

To identify factors for depression, sociodemographic data (such as age, gender, race, education level, income, and marital status) and clinical characteristics (type of psoriasis, presence of psoriasis arthropathy, anatomical site of psoriatic lesions, and comorbidities) were used as independent variables in a multiple logistic regression analysis.

Three variables have been found to be significantly associated with depression (p < 0.05): DLQI score, lower limbs' lesions and dyslipidaemia (Table 3).

**Table 3:** The association of clinical variables with depression by Multiple Logistic Regression analysis (n = 174).

Variables	Adjusted OR (95% CI)	P-value
Lesions on lower limbs		
No	1	
Yes	4.11 (1.134–14.896)	0.031
Dyslipidaemia		
No	1	
Yes	0.19 (0.049-0.755)	0.018
DLQI (score)	1.223 (1.112–1.345)	<0.001

## Discussion

The prevalence of depressive symptoms among participants is 8.5% with a mean score of 3.7 (SD 3.25). This prevalence is comparable to the overall national prevalence of depression, which ranges between 8 and 12%.^13^ The prevalence of depression among patients with psoriasis ranges between 6 and 62%.^14^ In comparison with similar studies using HADS for patients with psoriasis, this prevalence is less than a cross-sectional study performed in Singapore (15%)^15^ and a meta-analysis performed on this subject matter (23%).^14^ From the same meta-analysis, studies utilising Beck Depression Inventory (BDI) exhibited a higher prevalence of participants with depressive symptoms at 36%.^14^

One of the possible explanations for the difference in the prevalence of patients with depressive symptoms is the choice of questionnaire. HADS was chosen due to its feasibility of administration as it was originally intended for usage in medical settings. From a study among chronic obstructive pulmonary disease patients, it was suggested that the ability of HADS to differentiate between depressed and non-depressed patients among those suffering from chronic ailments was questionable, although it has been used regularly in various studies beyond its originally intended use in a general medical case mix pool in an outpatient setting.^16^ Thus, we can postulate that the instrument may not be disease-specific with the illness studied, as the concepts of body image, illness perception and stigma, which are related to disfiguring lesions may not be captured through the questionnaire. The absence of somatic symptoms in HADS may as well contribute to the reduced prevalence of depression in this study, as depression or anxiety may manifest themselves in physical symptoms.

There may be cultural influences that play a part in the relatively low prevalence of patients with depression in this study. In general, lifetime rates of depression are significantly much higher in the West as compared to Asian countries, and this difference has been theorised to be due to the better way of managing negative emotions in the Asian culture, which prevents the symptoms from escalating into a mood disorder.^17^ In a local study conducted among patients with end stage renal disease, only a small percentage suffered from depression, and this was associated with good social support received by patients.^18^ This favours the theory that Asians tend to have a collectivistic orientation that prioritises collective good over self, although it may be too premature to make such assumptions in general.^19^ While religious coping had been suggested as one of the means the Malay population deals with stress among cancer patients, there are studies that have shown that it may have a negative impact on a person's well-being.^20^ Due to the differences of chronic illnesses cited here, it is difficult to compare vis-à-vis with the subject matter of this research. Thus, further studies are needed to establish ways in which patients with psoriasis cope with this illness.

For anxiety symptoms, the prevalence obtained in this study is 16.9%, which is comparable to the research in Singapore (17%).^15^ In a systematic review that summarised studies using HADS-A to assess anxiety symptoms, 20–50% of patients with psoriasis showed significant levels of anxiety, and 7–16% were established to have an anxiety disorder.^21^ It has been suggested in this paper that anxiety symptoms may be due to poor self-esteem and social stigmatisation.

This study is unable to establish any association between sociodemographic data with depressive and anxiety symptoms. In a separate study conducted in Malaysia,^11^ stress was found to be highest among the Indian population with psoriasis but this study did not find any significant association between race and depressive symptoms, and the participants were predominantly Malays, making it difficult to arrive at any conclusion on this particular sociodemographic profile. The prevalence of participants who scored more than 10 for DLQI was 44.9% (n = 78) and this result is slightly higher than the 10-year review of the Malaysian Psoriasis Registry,^10^ which recorded a prevalence of 33.1% of patients with psoriasis with severe impairment.

In both correlational analysis and multivariate logistics regression, the DLQI score was the factor associated with both depression and anxiety, indicating that higher scores had contributed to anxiety and depressive symptoms. In fact, this was the only factor associated with anxiety symptoms. This is supported by many studies that found the association between poor quality of life^8^^,^^11^^,^^22^^,^^26^ in psoriasis patients leads to various psychological problems such as anxiety and depression.^3^^,^^6^^,^^7^^,^^14^^,^^15^^,^^21^^,^^24^ A study had found that the subjective measure of quality of life is a better predictor of depressive symptoms than objective measure of illness.^22^ The cosmetic disfigurement may lead a person with psoriasis to feel embarrassed, flawed and stigmatised, resulting in disruption of personal relationships, and daily, work-related, and leisure activities. The other factor that related to lowering the quality of life is the nature of this disease that leads to feelings of hopelessness of cure.^9^^,^^11^

Besides the DLQI score, presence of psoriatic lesions in the lower limbs and of dyslipidaemia were associated with depressive symptoms. While most studies have found an association between high psoriasis area severity index (PASI) scores, indicating a severe condition of psoriasis, and depressive and anxiety symptoms,^6^, ^7^, ^8^ some authors^23^, ^24^, ^25^ have argued that this objective severity measure fails to reflect the lesions that may appear on exposed parts of the body such as the face and hands, which may cause them to have a negative perception of their appearance. The association between lower limbs' lesions and depression may possibly be explained by pain that the patient experiences while walking. However, the results obtained in this study need to be interpreted with caution due to the rather simplified clinical assessment. A Polish research conducted to find the association between site of skin lesions with depression and anxiety revealed that lesions on head, neck, hands, and arms were highly associated with these psychological issues.^26^ Sharing a similar essence, this study and the Polish research took into consideration patients' own subjective evaluation of the site of psoriatic lesions, but the latter incorporated body surface area (BSA) as an objective measure of disease severity to complement the subjective reports.

Although the exact relationship between dyslipidaemia and depression in not clear in the context of patients with psoriasis, a study had shown that presence of depression is associated with reduction of HDL cholesterol and an increase of abdominal obesity over time.^27^ A meta-analysis had shown that the relationship between metabolic syndrome, including dyslipidaemia, and depression to be bi-directional, as metabolic syndrome itself may raise inflammatory cytokines that may contribute to the development of depression. Further, depression itself may cause metabolic syndrome through poor diet and influencing abdominal fat accumulation.

There are several limitations with this study. As it is a cross-sectional one, the disadvantages of such study design apply. The prevalence of patients with depression and anxiety in this study may not be the true representative of the population. Furthermore, there is an absence of a control group to compare with. The causal references are limited by the study design, and some results have to be interpreted with caution. This study did not take into consideration the medications that patients are prescribed with, as some of what they might be taking, such as prednisolone, methotrexate, and cyclosporine may precipitate depressive and anxiety symptoms as well.

## Conclusion

Psoriasis is a chronic skin disease that affects the psychological well-being and quality of life for those diagnosed with it. This study revealed that among the sampled population, there is a prevalence of patients who are affected by depression and anxiety, and these symptoms are correlated with a poor quality of life. Overall, this may impact in the recovery process of patients. Thus, screening for these symptoms among this population may be needed for those suspected of psychological issues and those who have poor quality of life. The findings may hopefully contribute to the understanding of psoriasis and its psychological sequelae, as well as paving the way for further research involving other underlying constructs, such as perceived body image and stigma.

## Recommendations

It is recommended that a study could be conducted to involve a control group to increase its external validity. Thus, comparison could be made from the findings between these different groups. As it is a cross-sectional study, the disadvantages of such study design apply. The causal references are limited by the study design, and some results have to be interpreted with caution. Clearly, the findings in this study indicate a need for further examinations on the other possible reasons for variations in associated factors related to depression and anxiety among psoriasis patients. Future studies should take into consideration the medications that patients are prescribed with, as some of what they might be consuming, such as prednisolone, methotrexate, and cyclosporine may precipitate depressive and anxiety symptoms as well.

## Source of funding

This research did not receive any specific grant from funding agencies in the public, commercial, or not-for-profit sectors.

## Conflict of interest

The authors have no conflict of interest to declare.

## Ethical approval

Ethical approval was obtained from the Human Research Ethics Committee (HREC) Universiti Sains Malaysia (USM) with the JePEM reference code of USM/JEPeM/18030167 dated August 7th, 2018 and from the Ministry of Health (MoH), Medical Research & Ethics Committee with the reference code NMRR-18-1487-42274 (IIR) dated February 12th, 2019.

## Authors contributions

SJSZ and RSB conceived and designed the study. AFA and YCH participated in data collection. RSB and AFA worked on analysis. AFA wrote the initial draft of the article. RSB, SZSJ, and YCH wrote the final draft of the article. All authors have critically reviewed and approved the final draft and are responsible for the content and similarity index of the manuscript.
